# Influences of Agents with a Self-Reputation Awareness Component in an Evolutionary Spatial IPD Game

**DOI:** 10.1371/journal.pone.0099841

**Published:** 2014-06-19

**Authors:** Chung-Yuan Huang, Chun-Liang Lee

**Affiliations:** Department of Computer Science and Information Engineering, School of Electrical and Computer Engineering, College of Engineering, Chang Gung University, Taoyuan, Taiwan, Republic of China; University of Maribor, Slovenia

## Abstract

Iterated prisoner’s dilemma (IPD) researchers have shown that strong positive reputations plus an efficient reputation evaluation system encourages both sides to pursue long-term collaboration and to avoid falling into mutual defection cycles. In agent-based environments with reliable reputation rating systems, agents interested in maximizing their private interests must show concern for other agents as well as their own self-reputations–an important capability that standard IPD game agents lack. Here we present a novel learning agent model possessing self-reputation awareness. Agents in our proposed model are capable of evaluating self-behaviors based on a mix of public and private interest considerations, and of testing various solutions aimed at meeting social standards. Simulation results indicate multiple outcomes from the addition of a small percentage of self-reputation awareness agents: faster cooperation, faster movement toward stability in an agent society, a higher level of public interest in the agent society, the resolution of common conflicts between public and private interests, and a lower potential for rational individual behavior to transform into irrational group behavior.

## Introduction

Reputation provides a foundation for game theorists to analyze ways that past behaviors of social participants affect the behaviors and strategies of iterated prisoner’s dilemma (IPD) game opponents. According to Nowak [1], the five mechanisms that promote cooperative behaviors are kin selection, direct reciprocity, indirect reciprocity, network reciprocity, and group selection. Reputation can be analyzed as a common form of indirect reciprocity based on knowing a player’s history with other players. It is a well-studied mechanism that sustains cooperation in evolutionary IPD games. For other examples see Fu *et al.* [2] and Wang *et al.* [3]. Using the game as a social interaction model, participants who always choose to cooperate with opponents can be described as having good reputations, but participants who always defect are viewed as having damaged reputations. Participants who establish good reputations tend to receive trust, praise, and other positive feedback from their partners; those with poor reputations do not. We believe that the combination of a positive reputation and accurate reputation evaluation system can encourage two parties to pursue long-term collaboration and to avoid falling into mutual defection cycles, even when faced with short-term sacrifices.

Reputation-related behaviors and strategies have meaning for online commerce. Web 3.0 is supporting a growing number of Internet platforms and commercial applications that use intelligent agent architectures in support of complex online tasks such as auto-bidding on auction websites, placing Internet stock transaction orders, and shopping for cheaper e-commerce products and services [4]–[6]. Web 3.0 researchers are therefore experimenting with artificial intelligence (AI) techniques to help intelligent agents “live” in Internet communities in ways that resemble how humans live in real-world communities [7]–[10]. However, because of Internet agent properties such as anonymity, mobility, and multiple identities [11], [12], the use of intelligent agents raises serious game-based and theoretical issues involving cooperation and defection scenarios and conflicts between public and private interests [13]–[15]. In their current form, intelligent agents do not have to worry about maintaining positive self-images, saving face, or being victims of acts of vengeance associated with fraudulent and defective behaviors commonly found in Web 3.0 e-commerce activities. Since they only care about private interests, they are unlikely to cooperate with other intelligent agents in support of group interests, thus increasing the potential for falling into cycles of never-ending mutual defection [16].

Several agent-based computational simulation researchers have shown that determining game strategies and behaviors based on an opponent’s reputation is an effective solution that may increase the desire to cooperate [2], [15], [17]–[26]. When one intelligent agent is required to cooperate with an unfamiliar agent to complete a task, a reputation rating system can have great utility in determining the unfamiliar agent’s trustworthiness [27], [28]. However, a clustering effect resulting in decreased public interest may occur if all agents in a system simultaneously search for other agents with good reputations. This seems inevitable, since agents with good reputations only want to work with other reputable agents. In contrast, agents lacking good reputations must spend a great deal of time performing partner searches because they are in the awkward position of being rejected by ideal partners, in many cases without self-knowledge of their poor or unspecified reputations [13], [29]–[34]. Further, if a reputable agent only cares about other agents’ reputations but lacks self-knowledge of its own reputation, it may try to maximize its private interests using behaviors that end up harming other agents, thus causing damage to its existing reputation. Accordingly, any multi-agent system with a reputation-rating scheme must contain a method so that agents interested in maximizing their private interests can exhibit concern for other agents as well as their own reputations.

Our proposed agent model is equipped with a self-reputation awareness component (SRAC) that learns and evolves during spatial IPD games involving two-dimensional social interaction networks. The SRAC agents in our model are capable of evaluating their behaviors based on a mix of public and private interest considerations, and of testing various solutions aimed at meeting and maintaining social standards. Self-reputation awareness helps new agents quickly learn that private interest maximization is best achieved via long-term cooperation with partners, which also serves to enhance their own reputations and to support their wishes for ideal partners in the future. In other words, agents with self-reputation awareness that show concern for their reputations are more likely to be self-adaptive, to evaluate their reputations based on their partners’ evaluations, and to determine the best strategies and behaviors for achieving both long- and short-term goals. According to our IPD simulation experiment results, as long as an artificial society has a small percentage of agents with this capability for self-reputation awareness, there will be faster cooperation, faster movement toward stability in an agent society, greater public interest in the agent society, resolutions of common conflicts between public and private interests, and decreased potential for rational individual behavior to change into irrational group behavior.

### Related Works

Self-awareness, a psychological process in which attention is directed at oneself [35], is a foundation for personality development and modification that affects all human behaviors. According to theorists, when humans achieve strong states of self-awareness, they tend to consider whether characteristics and behaviors such as personality, abilities, desires, needs, comportment, and values are appropriate [31], [36], [37]. Subsequent actions are thought to be more likely to reduce self-discrepancies and to meet inner identity standards established by important others, as well as societal and cultural values [38]. In other words, an intact sense of self-awareness supports a complete understanding of one’s own behavior in terms of right/wrong, good/bad, and value based on societal standards [39]. This capability is helpful for learning skills and adjusting strategies for interacting with others. Internally, one can recognize emotions, motivations, interests, and desires, increase self-identification, and achieve self-realization. Without this capability, one’s behaviors will often be triggered by strong momentary emotions without considering potential consequences [31]. Those individuals who are incapable of understanding the emotions and ideas of others are much more likely to expose their own shortcomings or to show off their strengths without contemplating the appropriateness of doing so.

In contrast to human models, the focus of learning and attention for intelligent agents has always been the external environment [40]. The world model gradually established during an agent’s learning process is a miniature of its external environment. The purpose of such a model is to maintain relationships between stimulation signals from external environments and behavioral reactions [41]. Based on physical environment and the presence of other agents, and using specific learning methods such as artificial neural networks, genetic algorithms, and fuzzy rule-based systems, agents continuously adjust their internal strategies, learn various skills [42], [43], and find problem solutions that satisfy user needs or fulfill assigned tasks [44], [45].

There are at least five advantages to equipping intelligent agents with a self-awareness capability: (a) compatibility with previous AI agent-learning frameworks, thereby supporting the expansion of existing cognitive structures so as to enhance agent learning outcomes and support searches for fast problem-solving strategies; (b) the introduction of self-consciousness so that agents, using mechanisms that connect external stimulation signals with behavioral reactions, can consider and integrate the mutual needs of or feedback from other agents that they interact with; (c) agent use of private and public self-consciousness for detecting its own behavioral reactions, differences, and discrepancies between internal and external standards, and for exploring means for improvement that may decrease such discrepancies, increase learning performance, and satisfy such standards; (d) support for understanding and recording the dynamic characteristics of their external environments, and in revising and adjusting internal standards or states accordingly; and (e) support for establishing artificial societies and agent cognitive and learning models that are similar to the ways that real societies operate [44], [46], [47].

### Spatial IPD Simulator and SRAC Agent Model

Our adaptive agent model contains a self-reputation awareness component (SRAC) based on a mix of social expectation strategies and a reputation evaluation procedure for resolving ongoing conflicts between public and individual private interests in an agent society. It is our belief that an awareness capability that allows agents to reflect on their self-reputations will result in more and faster collaborative behaviors and social benefits. To assess the effects of mixing SRAC and non-SRAC agents on the evolutionary dynamics of IPD games, we used the Java programming language to develop a general-purpose and extendable evolutionary spatial IPD simulator suitable for detailed numerical experimentation and classroom demonstrations. As shown in the screen-shot of Appendix S1, the IPD simulator is suitable for all common operating systems containing the Java virtual machine, including Linux, Mac OS X, and Windows. Executable files are available in a shared Google drive folder (https://drive.google.com/folderview?id=0B2C9hdWHlsqHbzNadVdGMGZxZkk&usp=sharing); for source code that matches specific research requirements, contact the corresponding author.

The simulation flow consists of four steps:
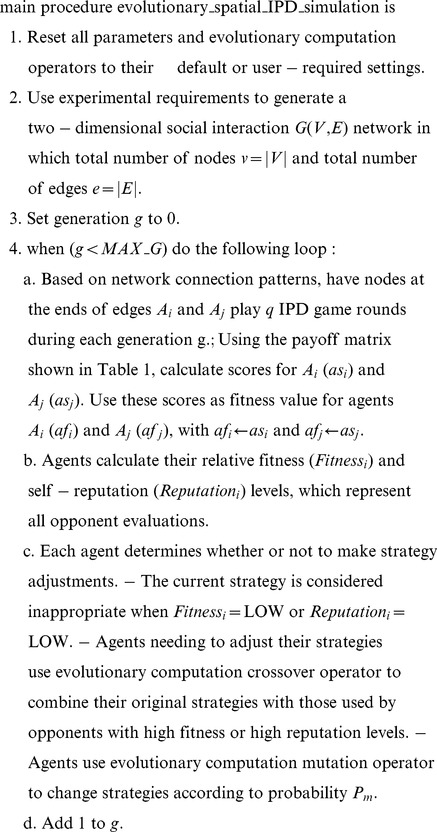



In Step 1, all parameters and evolutionary computation operators must be reset to default or user-required settings. The default settings allow first-time simulation users to quickly execute simple and understandable demonstrations. We categorized individual parameters as IPD game, social interaction network, or evolutionary computation. The first category includes the total number of interactions between an agent and its opponent (*q*), agent memory capacity (*c*), and agent strategy length (*l*). The social interaction network category includes the width (*W*) and height (*H*) of a two-dimensional social network, total number of nodes (*v*) and edges (*e*), neighborhood pattern (*P*), network type (*T*), and edge rewiring probability (*ρ*). The evolutionary computation category includes crossover rate (*P_c_*), mutation rate (*P_m_*), and total number of generations for each experiment (*MAX_G*). We used the following default values: *q* = 100, *c* = 1, *l* = 4, *W* = 50, *H* = 50, *v* = 2500, *e* = 10,000, *P* = Moore neighborhood and periodic boundary condition, *P_c_* = 0.7, *P_m_* = 0.01, and *MAX_G* = 100. The default configuration of the *P* neighborhood pattern parameter ensures that all nodes have equal numbers of neighboring nodes, and that each node establishes connections with its eight surrounding nodes to form tightly clustered groups.

Following initialization and parameter setting according to experimental requirements, a specific two-dimensional *W*×*H* social interaction network consisting of *v* nodes and *e* edges can be established according to the *T* parameter value. Each social interaction network node represents an IPD agent that is assigned a randomly generated memory-*c* deterministic strategy. Each edge represents a single IPD interaction relationship between two agents that are labeled as neighbors. Each IPD agent has an average of 2*e*/*v* neighbor opponents.

The *T* parameter can be set as either a cellular automata with high degrees of local clustering and separation, or a small-world network with a high degree of local clustering and low degree of separation. Cellular automata are widely used computational social science investigations of the large-scale outcomes of millions of small-scale events, and for creating visually striking patterns. Small-world networks, which are considered similar to human social networks, serve as the underlying foundations of social simulation models that are said to have high levels of reliability. To compare simulation results for the two network types, we stipulated that the numbers of nodes and edges in each must be equal. To satisfy this condition, if the *T* parameter is designated as small-world, the simulation is programmed to initially generate a two-dimensional cellular automata according to the *P* (neighborhood pattern) parameter configuration, and then to use a predetermined edge rewiring probability *ρ* (default: 1%) to determine whether or not individual edges must be rewired. If rewiring is necessary, either one of the two original nodes (one on each side of an edge) is discarded and replaced with a new, randomly selected node.

In Step 4a, for the sake of simplicity but without loss of generality, we used three IPD agent assumptions: (a) Agent *A_i_* has *n* opponents, meaning that opponents *O_i_* =  (*o_i,0_*, *o_i,1_*, …, *o_i,n−1_*)^g^ during generation *g*, with *o_i,j_* representing the *j*th opponent of *A_i_*. (b) Agent *A_i_* plays *q* IPD game rounds with each opponent during each generation. (c) The *af_i_* fitness value of agent *A_i_* equals the average of all payoffs received by that agent during rounds played within one generation. This value serves as an indication of its performance compared to others in the same agent population.

The IPD game payoff matrix used in Step 4a is shown in Table 1. As indicated, *R* = 3 represents the reward for mutual cooperation, *T* = 5 one party’s temptation to defect, *S* = 0 the “sucker’s payoff”, and *P* = 1 the punishment for mutual defection. Two conditions for generating a prisoner’s dilemma are *T*>*R*>*P*>*S* and 2*R*>*T*+*S*. The first guarantees that two rational agents will simultaneously betray each other after understanding that *T*>*R* and *P*>*S*, and therefore follow the second best choice, which is mutual defection (*P*, *P*). According to the second condition, prisoners cannot escape the same predicament by taking turns betraying each other–in other words, benefits for mutual betrayal are not as good as for mutual cooperation. Accordingly, each agent must rely on past behaviors to formulate strategies that optimize long-term benefits.

**Table 1:** IPD game payoff matrix.

		Player B	
		Cooperation (C)	Defection (D)
**Player A**	**Cooperation (C)**	R = 3**,** R = 3	T = 5**,** S = 0
	**Defection (D)**	S = 0**,** T = 5	P = 1**,** P = 1

Numbers in each cell are payoffs for both players, with player A’s payoff listed first in each pair.

The default strategy in our model is memory-1 deterministic, with agents remembering the behaviors of their opponents in preceding rounds. There are only four possible combinations: both cooperate (expressed as CC), one cooperates and the other defects (CD), one defects and the other cooperates (DC), and both defect (DD). Thus, the memory-1 deterministic strategy can be expressed as the four-value tuple (*S_cc_*, *S_cd_*, *S_dc_*, *S_dd_*): if an agent’s memory of the preceding round is CC, then it will choose *S_cc_* when responding to an opponent. Since responses are limited to either cooperation (C) or defection (D), a memory-1 deterministic strategy consists of 16 (2^4^) possible combinations of moves. Among these, *S_0_* =  (C, C, C, C) is known as the “yes-man” (YM) strategy, *S_5_* =  (C, D, C, D) the “tit-for-tat” (TFT) strategy, *S_6_* =  (C, D, D, C) the “win-stay, lose-shift” (WS/LS) strategy, and *S_15_* =  (D, D, D, D) the “scoundrel” (S) strategy. These four strategies have attracted considerable research interest. The WS/LS strategy applies Pavlovian psychological theory in proposing that an agent will adhere to one strategy until its income goes below a threshold, after which it switches to the opposite strategy [48]. In the TFT strategy, an agent always chooses cooperation during the first round of a game, and then imitates its opponent’s strategy in subsequent rounds.

In Step 4b, each agent initially uses the evaluation algorithm described in Appendix S2 to give its opponent a relative reputation score at the end of each generation, based on the mean and standard deviation of the number of cooperative moves made by its opponents during one generation. By applying this relative reputation evaluation algorithm, the two algorithms proposed in Appendix S3 can be used to respectively compute an agent’s relative fitness and self-reputation levels in the contexts of its opponents.

**Figure 1:**
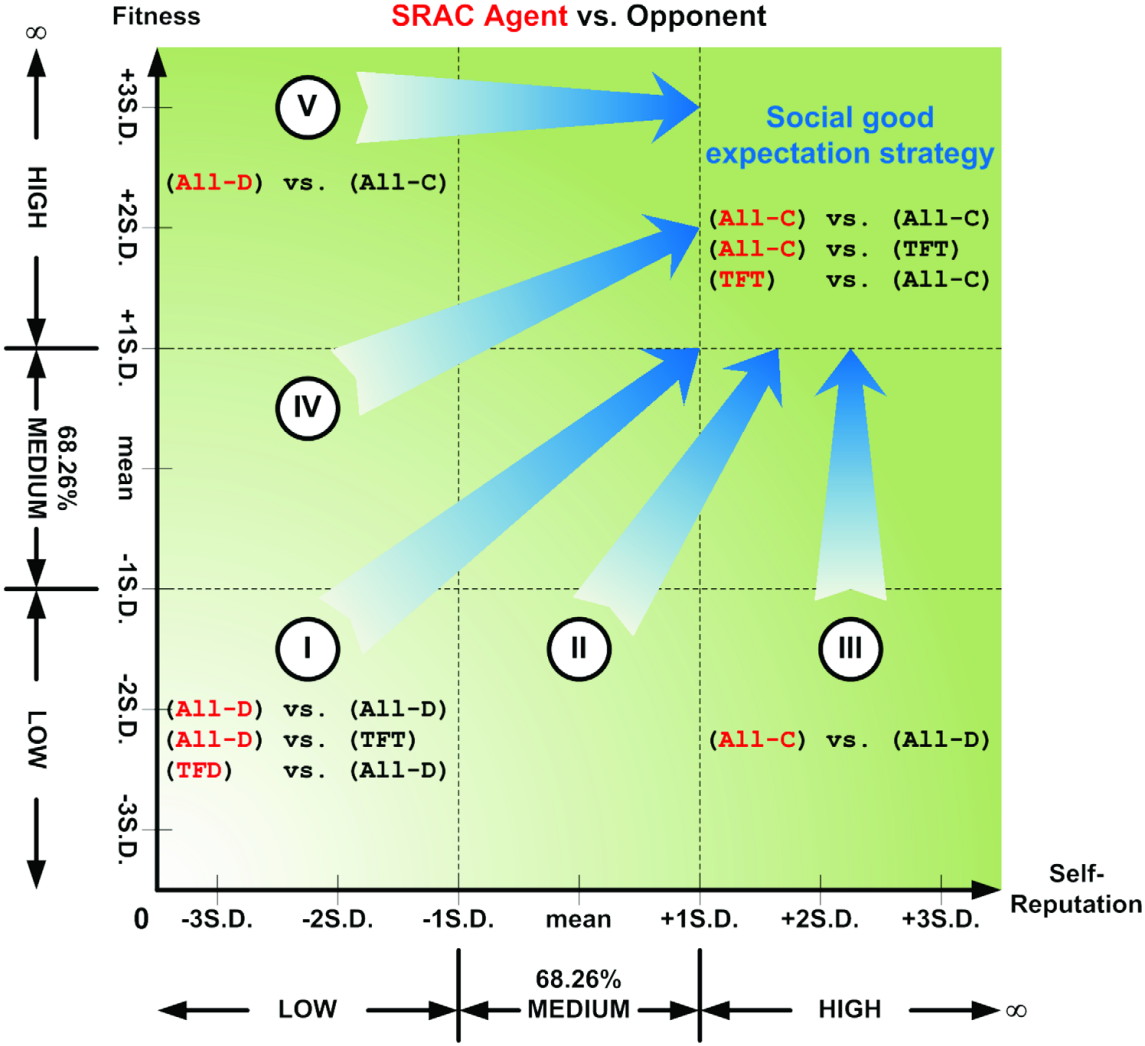
Agent fitness scores plotted against a self-reputation index matrix.

As shown in Figure 1 and Step 4c of the pseudo-code of our IPD simulation, fitness and self-reputation levels are categorized as high, medium, or low, resulting in nine possible interaction types between an SRAC agent and its opponent. As an example, a SRAC agent with a high degree of fitness and low degree of self-reputation usually adheres to an always-betray or similar “villain” strategy that cannot produce a higher public good value, since it diminishes the ability of other agents to pursue their own interests. Therefore, SRAC agents must be taught that an always-betray strategy will negatively affect their reputations. By referring to and learning from their opponents’ positive performance strategies that conform to social expectations, SRAC agents can achieve higher levels of fitness and self-reputation.

## Results and Discussion

**Figure 2:**
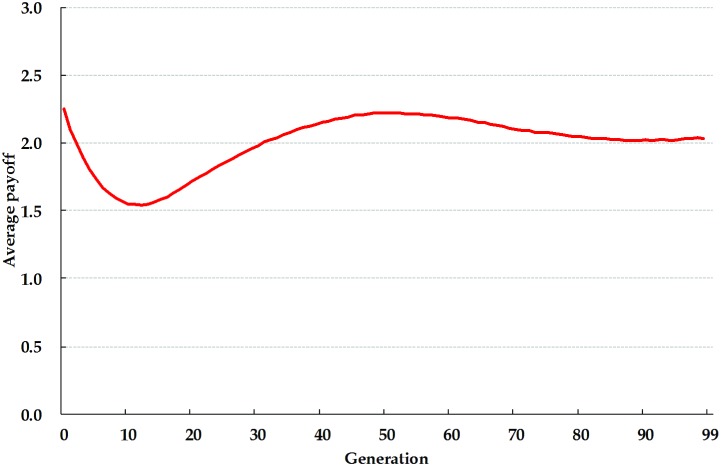
Average payoffs for all agents in cellular automata without adding SRAC agents.

**Figure 3:**
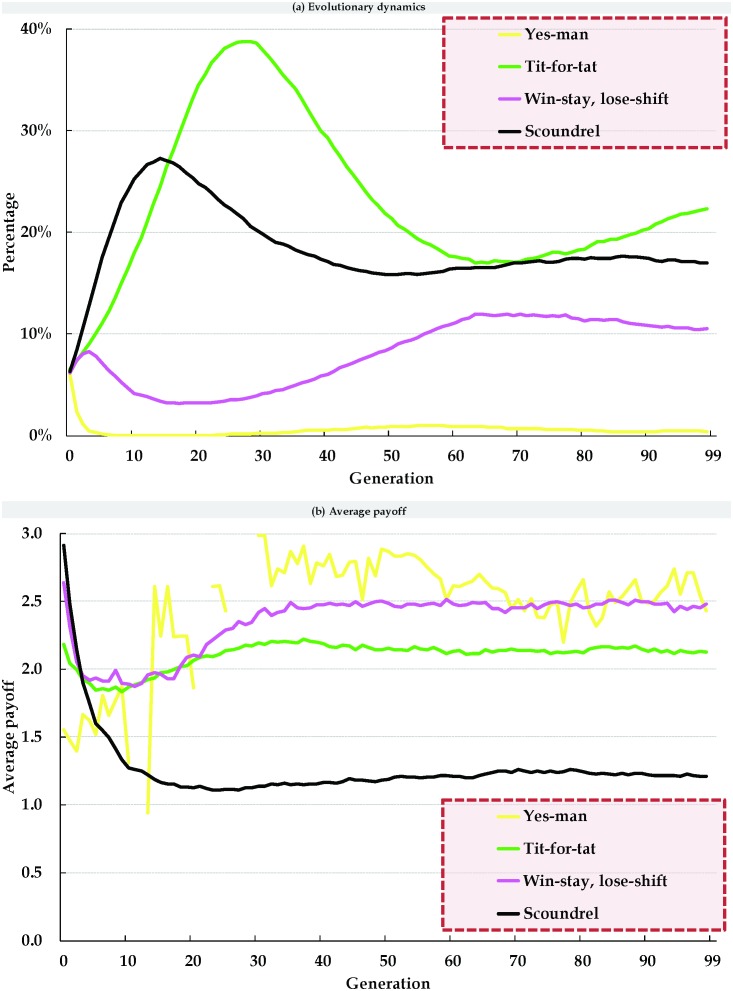
Evolutionary dynamics and average payoffs for four IPD strategies in cellular automata without adding SRAC agents.

**Figure 4:**
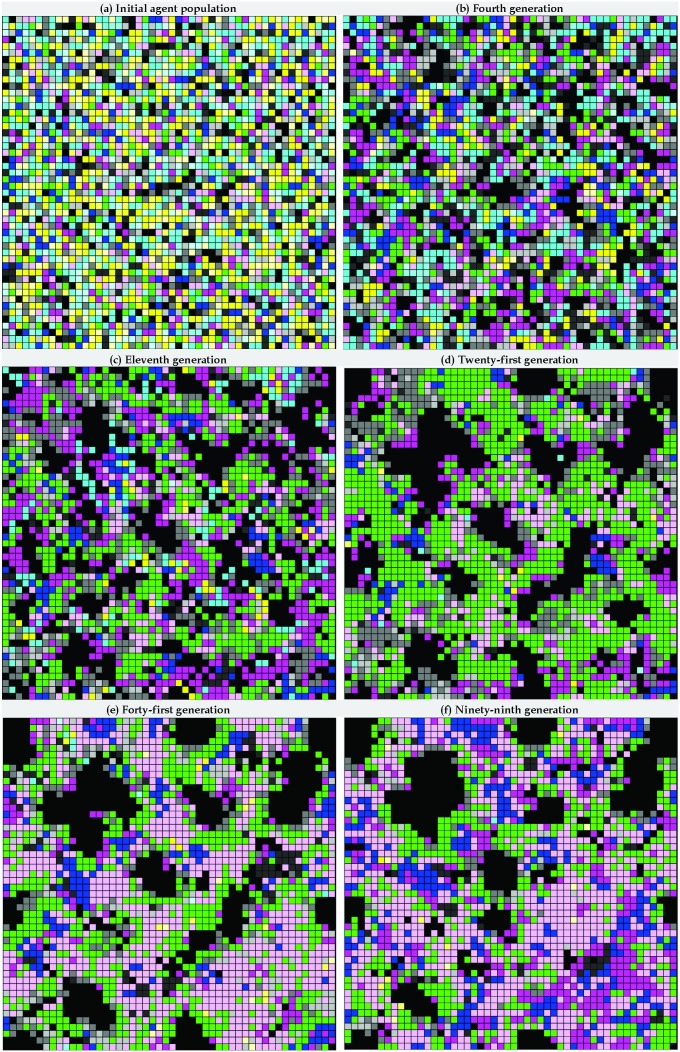
Spatial distributions of 16 memory-1 deterministic IPD strategies in cellular automata without adding SRAC agents.

Our first task was to analyze the results of IPD game simulations using cellular automata and without adding any SRAC agents (Figs. 2, 3 and 4). The first 99 generations can be divided into five stages based on the evolutionary dynamics and spatial distributions of agent-adopted strategies. During the first stage (generations 0–3), our proposed model starts with a pool of randomly generated strategies adopted by individual agents being evenly distributed throughout the cellular automata (Fig. 4a). During the second stage (4–10), agents tend to give in to the temptations of maximizing their private interests and use the S strategy. As stated earlier, when a majority of agents adopt that strategy, the entire community eventually enters a cycle in which overall and individual private benefits rapidly decrease (Figs. 2 and 3b). In cellular automata, if the majority of an agent’s adjacent neighbors adopt the S strategy, then the agent in the center is forced to adopt the same strategy in order to survive (Figs. 4b and 4c).

During the third stage (11–20), agents wanting to move away from the S strategy are likely to move toward a TFT strategy (Fig. 3a). In addition to confronting S strategy agents, this action also supports cooperation with agents who adopt either the YM or TFT strategies. The spatial clustering effect is also known as network reciprocity [1], [49]. Network reciprocity is receiving attention from physics researchers [24]–[26], [50]–[54]. Figures 4c and 4d illustrate a scenario in which TFT strategy agents gradually increase in number and cluster in a manner that surrounds and restricts agents who adopt the S strategy.

The number of TFT strategy agents declines during the fourth stage (21–40). Due to an asymmetry problem involving memories of previous encounters, these agents start to defect and stop trusting each other, resulting in less clustering over large areas. However, as shown in Figures 4d and 4e, some TFT strategy agents continue to surround S strategy agents to ensure that the latter do not expand to the point of overwhelming the former. Note also that as clusters of TFT strategy agents start to break up and decrease in size, the number of agents that adopt the WS/LS strategy increases (Fig. 3a) [1]. Since WS/LS strategy agents do not have asymmetric memory problems regarding previous encounters (which increases the potential for breaking promises), and since those same agents generally move toward mutual collaboration, their numbers and tendency to cooperate gradually increase.

During the fifth stage (41–100), strategy evolution enters a state of “dynamic stability”–a term we use to describe a long period of repetition. Within clusters of WS/LS strategy agents, the number of YM strategy agents gradually increases (Figs. 4e and 4f). Agents who adopt either the YM or WS/LS strategies interact in ways that benefit both sides. However, in reaction to this increase, some agents take advantage of the situation by reverting to the S strategy, which reduces (and in some cases eliminates) clusters of agents that adopt the all-cooperation strategy. This scenario, which is often found in human societies, increases the potential for damage from internal mutation and external invasions.

**Figure 5:**
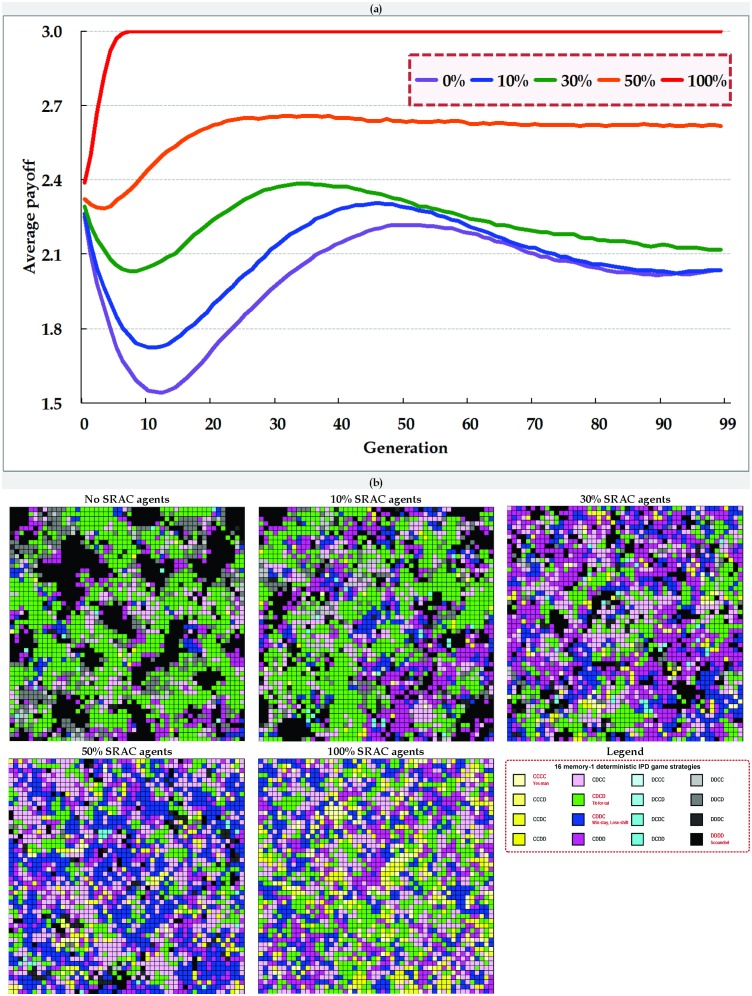
Comparisons of (a) average payoffs and (b) spatial distributions of 16 memory-1 deterministic game strategies at the twenty-first generation triggered by the addition of different percentages of SRAC agents in cellular automata.

Average payoff curves from our IPD simulations using 0% (baseline), 10%, 30%, 50% and 100% SRAC agents are shown in Figures 5 (cellular automata) and 6 (small-world network). Initial parameter settings were identical. As indicated by the red average payoff curves in the two figures, the overall network community clearly benefited when all agents possessed the capacity for self-reputation awareness, with a state of dynamic stability achieved within very few generations. However, since such a situation is not possible in the real world, we focused on the effects of adding a small number of SRAC agents to an otherwise unaltered environment. According to the blue (10%) and green (30%) average payoff curves, adding a small number of SRAC agents exerted a significant influence, regardless of social interaction network type. Specifically, they suppressed growth in the number of agents who adopted the S strategy, prevented the initiation of a cycle in which all agents expressed betrayal and retaliatory behaviors, and helped resolve conflicts between society-wide benefits and individual private interests so that cooperation gained acceptance as mainstream behavior.

**Figure 6:**
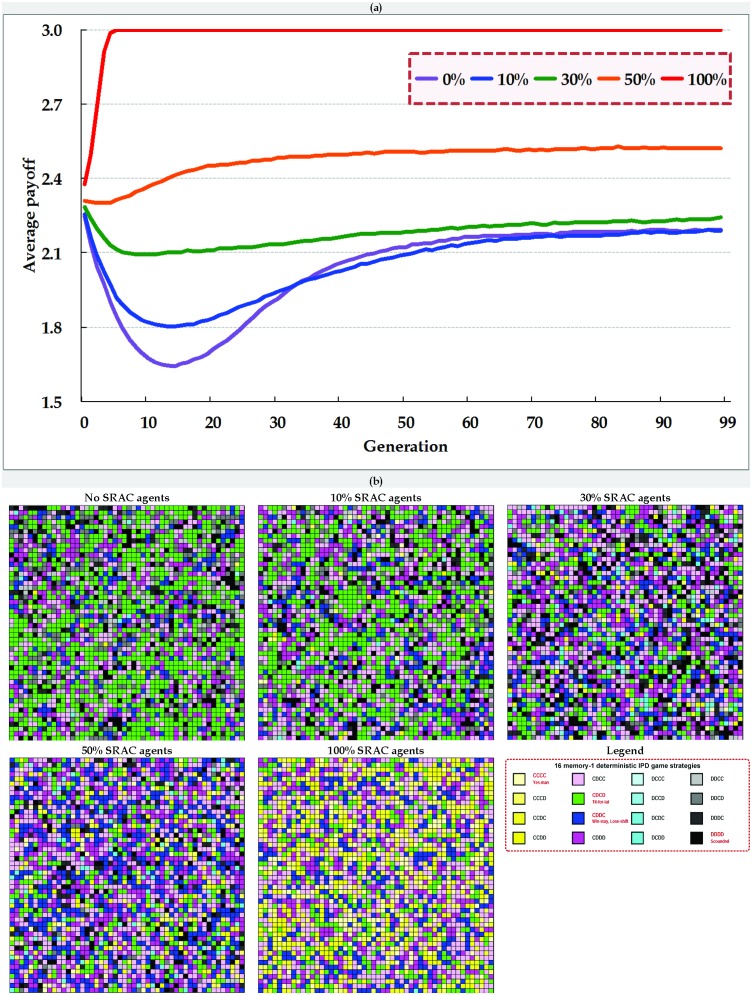
Comparisons of (a) average payoffs and (b) spatial distributions of 16 memory-1 deterministic game strategies at the twenty-first generation triggered by the addition of different percentages of SRAC agents to small-world networks.

The average payoff curves in Figures 5 and 6 are similar because small-world networks contain many random long-distance shortcuts that reduce network separation. There are at least two reasons for a lack of strategic clustering: these shortcuts produce very low degrees of separation (approximately log *v*, with *v* representing the total number of nodes), and they significantly increase complexity in terms of agent interactions and indirect influences. Note that the influence of a single game strategy can result in increased evolutionary diffusion and the increased containment of other agents. Combined, these factors accelerate the movement toward dynamic stability.

Agents who follow a YM strategy are the most likely to be taken advantage of by agents who use tactics associated with a S strategy. In contrast, TFT strategy agents find it easy to cooperate with YM and to attack S strategy agents. However, due to memory asymmetry problems regarding previous encounters, interacting TFT strategy agents may express negative behaviors such as breaking promises for an extended time period. Finally, we found that WS/LS strategy agents tended to change their behaviors as soon as benefits from doing so became obvious.

**Figure 7:**
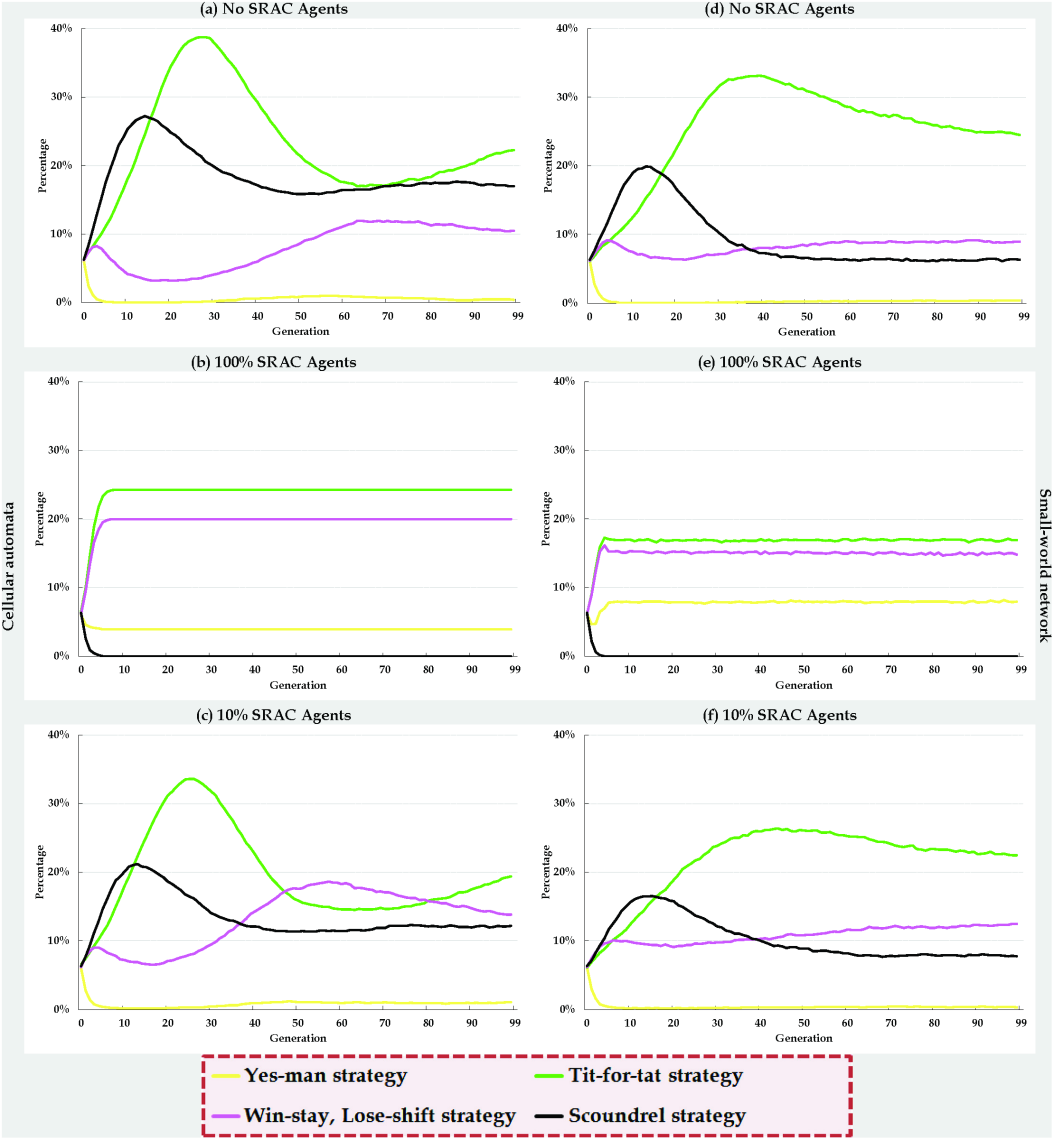
Evolutionary dynamics of four IPD strategies in cellular automata (a, b, c) and small-world networks (d, e, f).

**Figure 8:**
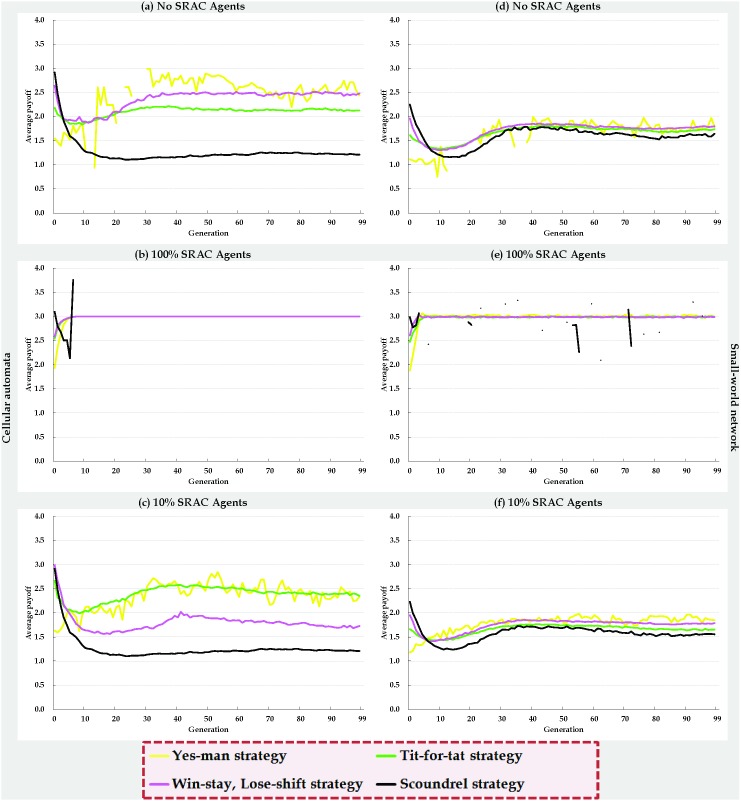
Average payoffs for four IPD strategies in cellular automata (a, b, c) and small-world networks (d, e, f).

We analyzed the evolutionary dynamics (Fig. 7) and average payoffs (Fig. 8) of the four strategies in terms of three SRAC agent mixes–0% (Figs. 7a, 7d, 8a, and 8d, control group), 100% (Figs. 7b 7e, 8b, and 8e), and 10% (Figs. 7c, 7f, 8c, and 8f); all other parameter settings were identical. Using cellular automata with 0% SRAC agents resulted in roughly equal numbers of agents adopting each of the four strategies at the beginning of every simulation (Fig. 7a). After three generations, the number of agents adopting the S strategy increased rapidly, and the number of agents adopting the YM or WS/LS strategies decreased slightly. Agents adopting the TFT strategy emerged when the number of S strategy agents reached a certain threshold. As described earlier, they confronted and suppressed agents adopting the S strategy, and collaborated with agents adopting the YM and WS/LS strategies. After twenty generations, the number of agents adopting the TFT strategy surpassed the number of agents adopting the S strategy, resulting in a sharp decrease in agents adopting the S strategy. The number of agents adopting the WS/LS strategy steadily increased after thirty generations; after sixty generations, the number of agents adopting the TFT strategy fell below the number of agents adopting the S strategy, and the simulated agent society entered a state of dynamic stability. The numbers of agents adopting the WS/LS and YM strategies did not change, and a balance was achieved in the growth and decline of agents adopting the S and TFT strategies.

As shown in Figure 7d, early evolutionary growth and decline rates for all four strategies in two-dimensional small-world networks with 0% SRAC agents were similar to those shown in Figure 7a. After thirty generations, the number of agents adopting the S strategy reached a saturation point and remained at a fixed number that was significantly higher than that observed for the cellular automata. Due to the small-world network’s low degree of separation characteristic, the numbers of agents adopting each of the four strategies reached a state of dynamic stability between the fiftieth and sixtieth generations.

Figure 7c presents data on simulations involving cellular automata and the 10% addition of SRAC agents. Compared to Figure 7a (0% SRAC agents), the peak number of agents adopting the S strategy was not as great–a 150-agent difference. Figures 7d and 7f illustrate data for 0% and 10% additions of SRAC agents, respectively; here the difference in the peak number of agents adopting the S strategy was 60. Note also that following the 10% addition of SRAC agents, the number of agents adopting a WS/LS strategy surpassed the number of agents adopting the S or TFT strategies during generations 47 through 80 (Fig. 7c), but after the 80th generation those agents adopting the WS/LS strategy could not successfully resist agents adopting the S strategy, even though their numbers had increased. As a result, the number of agents adopting the WS/LS strategy started to decline to a stable level.

Figure 7b presents data for a cellular automata consisting of 100% SRAC agents. Since S agents quickly discovered that their strategy was inappropriate for fulfilling social expectations, during the early evolutionary stages they all used their self-adjustment mechanisms to adopt other strategies to meet the expectations of adjacent agents. Starting at the third or fourth generation, the number of agents adopting the S strategy dropped to zero, and no new S strategy-adopting agents emerged for the rest of the simulation. The number of agents adopting the other three strategies also quickly stabilized without additional changes. Again, all parameters in Figures 7b and 7e were identical; the evolutionary dynamics of the four strategies in the two types of social interaction networks were also virtually identical. The only significant difference was the presence of random long-distance shortcuts in the two-dimensional small-world network. Due to increased sensitivity, even small changes in a single agent’s strategy were capable of influencing the entire network. However, due to the low degree of separation characteristic of small-world networks, a new state of dynamic stability was quickly reestablished.

## Conclusion

In this paper we described our proposal for a self-reputation awareness model in which agents are given the ability to calculate and interpret their self-reputation levels, and to adjust their IPD game strategies accordingly. Our primary conclusions are (a) the model successfully encourages strategy adjustments to achieve an optimum balance between self-reputation and private interests, thus increasing the likelihood that an agent will suppress its betrayal behavior and defection strategy in order to increase cooperation with other agents; and (b) compared to other models, overall cooperative behavior in our proposed model is likely to emerge much faster.

Our proposed SRAC agent model incorporates numerous features taken from AI, cognitive psychology, economics, and the social/behavioral sciences. AI researchers have generally overlooked the learning processes through which individuals enact self-awareness mechanisms. Based on our experimental results, we believe that integrating a self-reputation awareness component into agent architectures not only brings the behaviors and interaction patterns of agents into closer agreement with those of real people, but also provides a novel agent architecture to help agent-based simulations more accurately reflect actual societal operations. It is our hope that this self-reputation awareness component will support the efforts of smart object researchers interested in improving internal cognition and external learning capability in intelligent agents. In terms of cognitive psychology, our proposed SRAC agents can utilize personality traits to enhance their self-understanding and self-identity, thus promoting self-realization. The model also offers a novel approach to the IPD game: as long as a small number of SRAC agents are added to an IPD scenario, public good/private interest conflicts can be resolved, agent cooperation can be increased, and overall societal benefits can be enhanced. Finally, in terms of social/behavioral sciences, observing clustering behaviors allows for greater understanding of how self-reputation awareness can influence evolutionary dynamics and average payoffs in artificial agent societies.

## Supporting Information

Appendix S1 **User interface for our evolutionary spatial IPD simulator.**
(DOCX)

Appendix S2 **Pseudo-code to evaluate the relative reputation scores of the agent’s opponents.**
(DOCX)

Appendix S3 **Pseudo-code to compute the agent’s relative fitness and self-reputation levels.**
(DOCX)
