# Supplementary figures and images for: Influences of Agents with a Self-Reputation Awareness Component in an Evolutionary Spatial IPD Game
Source: PLoS One. 2014 Jun 19;9(6):e99841. doi: 10.1371/journal.pone.0099841 (PMC4063756; doi:10.1371/journal.pone.0099841)

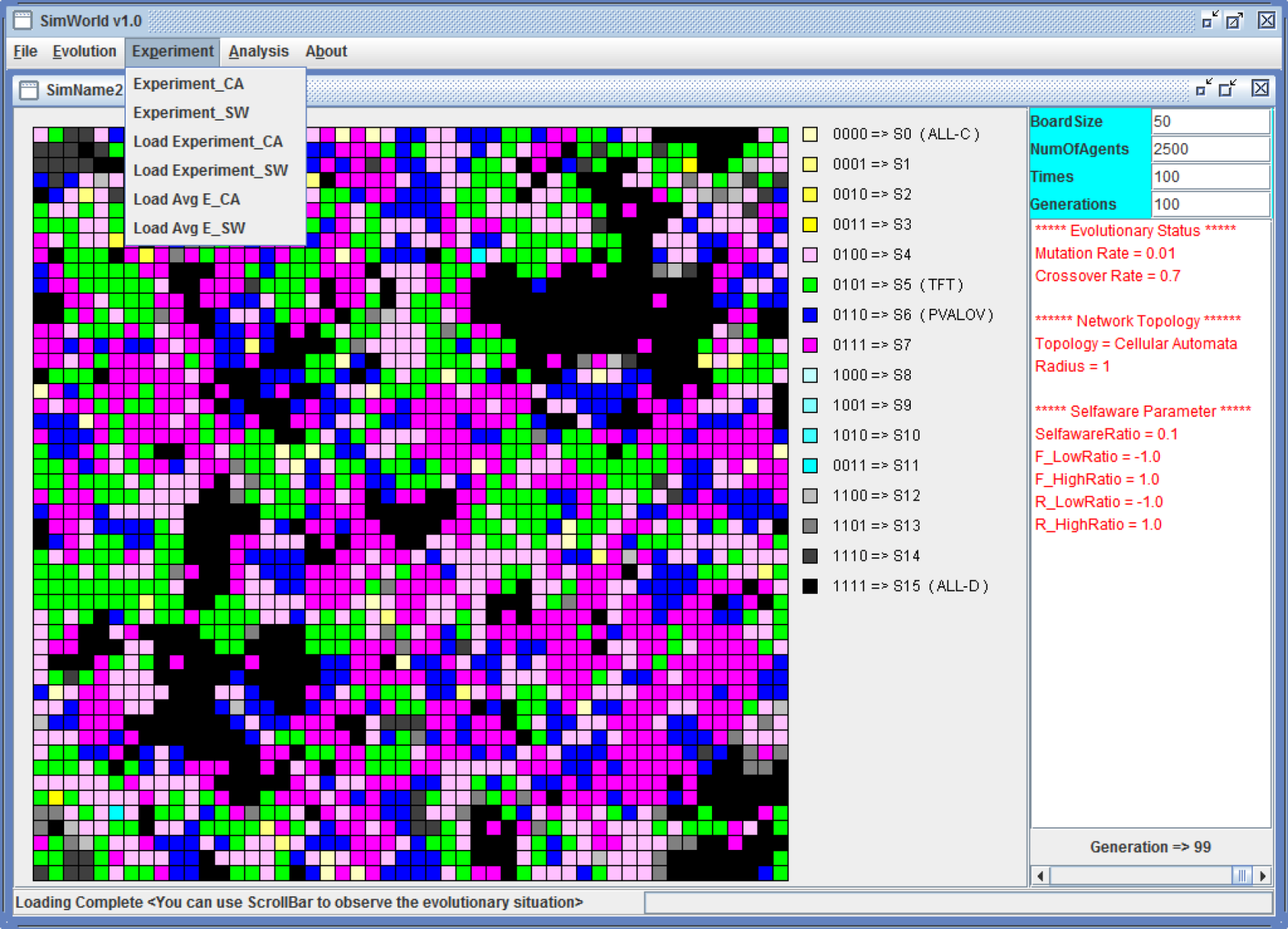

Supplement: Appendix S1 — User interface for our evolutionary spatial IPD simulator. (DOCX) [file pone.0099841.s001.docx]
