# Supplementary material for: Influences of Agents with a Self-Reputation Awareness Component in an Evolutionary Spatial IPD Game
Source: PLoS One. 2014 Jun 19;9(6):e99841. doi: 10.1371/journal.pone.0099841 (PMC4063756; doi:10.1371/journal.pone.0099841)
Supplement: Supplementary file 2 [file pone.0099841.s002.docx]

function evaluate_the_relative_reputation_scores_of_the_agent's_opponents (agent A_i) is

1. At the end of generation g, $C_i = (c_{0,i}, c_{1,i}, \ldots, c_{n-1,i})^g$, with $c_{j,i}$ representing the number of times that opponent $o_{i,j}$ makes a cooperative move during an IPD game with agent A_i in generation g.
2. Calculate the average value (avg_i) and standard deviation (std_i) of the numerical sequence C_i.
3. Compute the relative reputation value $r_{i,j}$ of agent A_i's opponent $o_{i,j}$ as $\left|(c_{j,i} - avg_i)/std_i\right|$.
4. Return the relative reputation list $R_i = (r_{i,0}, r_{i,1}, \ldots, r_{i,n-1})^g$ of agent A_i's opponents O_i in generation g.
