# Supplementary material for: Influences of Agents with a Self-Reputation Awareness Component in an Evolutionary Spatial IPD Game
Source: PLoS One. 2014 Jun 19;9(6):e99841. doi: 10.1371/journal.pone.0099841 (PMC4063756; doi:10.1371/journal.pone.0099841)
Supplement: Supplementary file 3 [file pone.0099841.s003.docx]

function compute_the_agent's_relative_fitness_level (agent A_i) is

1. Agent A_i collects fitness value data for all opponents O_i and creates the numerical sequence $OF_i = (f_0, f_1, \ldots, f_{n-1})^g$ with length n, f_j represents the fitness value of opponent $o_{i,j}$.
2. Calculate the average value (OF_avg_i) and standard deviation (OF_std_i) of numerical sequence OF_i.
3. $Fitness_i$ ← MIDDLE.
4. if agent A_i's fitness value af_i is less than (OF_avg_i - OF_std_i), then
   $Fitness_i$ ← LOW,
   else if agent A_i's fitness value af_i is greater than (OF_avg_i + OF_std_i), then
   $Fitness_i$ ← HIGH.
5. return $Fitness_i$.

function compute_the_agent's_relative_reputation_level (agent A_i) is

1. Agent A_i ask all $o_{i,j}$ opponents about their evaluations of its reputation.
   - In the numerical sequence $AR_i = (r_{0,i}, r_{1,i}, \ldots, r_{n-1,i})^g$, $r_{j,i}$ represents opponent $o_{i,j}$'s evaluation of agent A_i's reputation.
   - The information is used to calculate the average value (ar_i) of numerical sequence AR_i; the resulting ar_i represents agent A_i's average reputation in group O_i.
2. Agent A_i collects reputation information for opponents O_i from all other opponents by calling the evaluate_the_relative_reputation_scores_of_the_agent's_opponents function n times, and applies it to creates the numerical sequence $OR_i = (r_{0,1}, \ldots, r_{0,n-1}, r_{1,0}, \ldots, r_{1,n-1}, \ldots, r_{n-1,0}, \ldots, r_{n-1,n-2})^g$ with length $n \times (n - 1)$, $r_{j,k}$ represents opponent $o_{i,j}$'s evaluation of opponent $o_{i,k}$'s reputation.
3. Calculate the average value (OR_avg_i) and standard deviation (OR_std_i) of numerical sequence OR_i.
4. $Reputation_i$ ← MIDDLE.
5. if agent A_i's reputation ar_i is less than (OR_avg_i - OR_std_i), then
   $Reputation_i$ ← LOW,
   else if agent A_i's fitness value ar_i is greater than (OR_avg_i + OR_std_i), then
   $Reputation_i$ ← HIGH.
6. return $Reputation_i$.
